# Optimizing Stability and Performance of Silver-Based Grating Structures for Surface Plasmon Resonance Sensors

**DOI:** 10.3390/s23156743

**Published:** 2023-07-28

**Authors:** Pongsak Sarapukdee, Christian Spenner, Dirk Schulz, Stefan Palzer

**Affiliations:** Department of Electrical Engineering and Information Technology, Technical University Dortmund, Friedrich-Wöhler-Weg 4, 44227 Dortmund, Germany; pongsak.sarapukdee@tu-dortmund.de (P.S.);

**Keywords:** plasmon resonance, microfabrication, grating coupler, simulation model, label-free, protective layer, biosensors, silver, refractive index

## Abstract

The use of surface plasmon resonance sensors allows for the fabrication of highly sensitive, label-free analytical devices. This contribution reports on a grating coupler to enable surface plasmon resonance studies using silver on silicon oxide technology to build long-term stable plasmonic structures for biological molecule sensing. The structural parameters were simulated and the corresponding simulation model was optimized based on the experimental results to improve its reliability. Based on the model, optimized grating nanostructures were fabricated on an oxidized silicon wafer with different structural parameters and characterized using a dedicated optical setup and scanning electron microscopy. The combined theoretical and experimental results show that the most relevant refractive index range for biological samples from 1.32–1.46 may conveniently be covered with a highest sensitivity of 128.85°/RIU.

## 1. Introduction

Surface plasmon resonance (SPR) sensors provide highly sensitive, label-free detection with a fast response time and an efficient use of samples. SPR techniques form the basis for a multitude of biosensor studies that may be applied in various areas. It has shown significant advances as an essential tool for real-time monitoring of the interaction of different materials without using label molecules [1]. Recent developments in biosensors have included studying the interaction of biomolecules, such as enzymes [2], proteins [3], drugs [4], cells [5], DNA [6], viruses [7], and antigen and antibody interaction [8]. Most SPR-based sensor setups use a prism to excite surface plasmon polaritons (SPP) within the metal–dielectric material interface by coupling the electromagnetic wave to the collective electron oscillations. The sensitivity of the oscillations changing at the boundary is then used as a transducer to probe the medium’s properties.

Even though the prism SPR figuration offers an appropriate coupling efficiency for SPP excitation, it has limitations with regards to its potential for miniaturization and microfabrication. These drawbacks may be overcome by employing diffraction gratings to excite surface plasmons, since this approach is particularly suited for integration into small systems using the microsystem’s technology.

**Figure 1:**
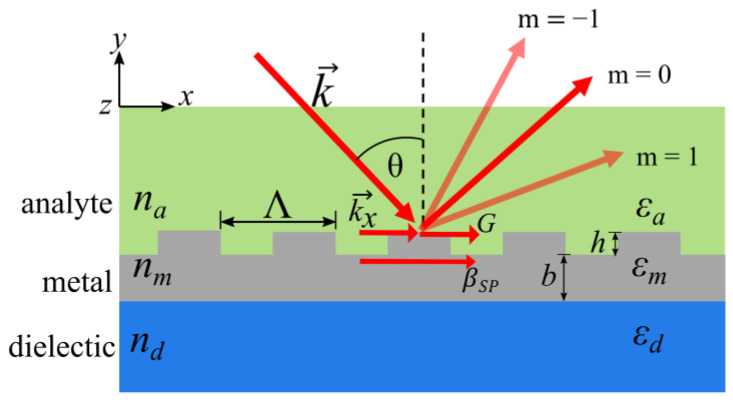
An incident beam with wave vector *k* under an angle θ with the surface is diffracted in different orders *m* according to the Bragg condition. In the case of the resulting diffracted wave vector coinciding with the wave vector of a surface plasmon, the latter may be excited and light energy can be transferred to the surface plasmon wave. In this case, the reflected beam intensity (*m* = 0) diminishes.

Surface plasmons may be excited by electromagnetic waves with wavelength λ in the visible range, which allows for using low-cost, standardized optical components to build an SPR system. In order to excite a surface plasmon wave, an incident light field must be coupled to the oscillations of the free electrons of the metal, thereby initiating the wave propagation. In grating structures with spatial period Λ and a corresponding grating vector $G=\frac{2\pi }{\Lambda }$, this is oftentimes accomplished via a diffracted beam, which enables matching to the surface plasmon parameters. Figure 1 depicts a schematic drawing of grating coupling introducing the relevant parameters of grating-coupled SPR sensors with angular sensing.

Efficient coupling may be achieved by using transverse magnetic (TM) polarized light to illuminate the grating under an angle of incidence of θ such that it leads to matched wave vectors of the surface plasmon wave $k_{sp}=\beta_{SP}=\frac{2\pi }{\lambda }\sqrt{\frac{\epsilon_{a}\epsilon_{m}}{\epsilon_{a}+\epsilon_{m}}}\;$ and the diffracted wave $k_{a}=\frac{2\pi }{\lambda }n_{a}sin\theta +m\frac{2\pi }{\Lambda }\;$. Here, $\epsilon_{a}$ represent the analyte permittivity and $\epsilon_{m}$ represents the metallic permittivity. The refractive index of the analyte is denoted as $n_{a}$, and the and *m* represents the grating’s diffraction order [9]. These considerations allow for calculating the coupling angle:

(1) $$\theta =sin^{-1}\left(\frac{1}{n_{a}}\left(\sqrt{\frac{\epsilon_{m\;}\epsilon_{a}}{\epsilon_{m}+\epsilon_{a}}}-m\frac{\lambda }{\Lambda }\right)\right)$$

This coupling leads to a reflected wave in the zero-order angular spectrum, depending on the coupling efficiency. The irradiation of the grating at different angles produces a series of diffracted waves.

Previous efforts [10,11] have demonstrated the possibility to combine the electrical characterization and grating detection in a single chip [12]. Additionally, more sophisticated systems promising an even better performance are being investigated [13,14]. Nonetheless, a limited number of SPR grating studies are concerned with experimental studies due to the challenging fabrication processes, which require a high level of control for the process parameters. This is particularly true for silver-based structures, even though silver provides the highest sensor performance potential.

Therefore, this study reports on the investigation of a grating-coupled SPR using a silver-based structure as the central building block and combines the experimental results with a dedicated simulation model to reliably simulate the coupling behavior. This work has focused on silver because it features the highest sensitivity of common plasmonic materials. Silver is known to be the best-performing choice at optical frequencies because of low losses in the visible spectrum for incident wavelengths [15]. However, it is prone to oxidation and degradation during sensor operation. While using gold as plasmonic material prevents oxidation of the structures, silver has been chosen due to its superior plasmonic properties, cost-effectiveness, and potential for future mass production. To address this problem, a protective layer has been utilized to mitigate oxidation and degradation. To study the sensitivity and stability, as well as the ideal technological process parameters, grating structures were fabricated on an oxidized silicon wafer with varying parameters, such as the layer thickness, grating period, grating width, and grating height. For each set of experimental parameters, the plasmon properties were determined by identifying the plasmon excitation angle. The results were used as reference data to feed back into the dedicated simulation model and demonstrate the suitability of the technology for the refractive index range of 1.32–1.46, which is the most relevant range for biological molecules’ sensing applications [16]. The performance using actual samples, as well as the stability of the coupling structures were investigated and a solution to enhance the long-term stability is presented.

## 2. Materials and Methods

In order to enable efficient, iterative optimization of grating structures and the corresponding simulation model, a complete development cycle entailing fabrication, characterization, and simulation was implemented within this work.

**Figure 2:**
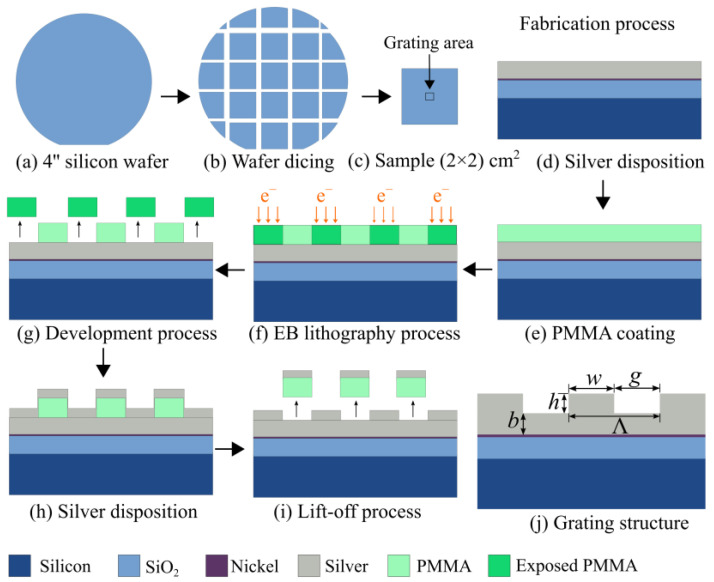
Grating fabrication process and grating geometry: (**a**) A 500 µm thick, thermally oxidized silicon wafer was used as the starting point of the process. (**b**) The wafer was cut in equal squares of (2 × 2) cm². (**c**) The grating area used to realize the grating structure was a square with 1.2 mm². (**d**–**j**) The grating structure was manufactured using silver as the material. To facilitate adhesion with SiO_2_, Ni was sputtered onto the substrate first. Using PMMA as the e-beam resist, the grating structure was fabricated with periodicity Λ.

The process to produce the silver grating structures is based on a 4″ Si wafer with a front- and backside finish of 100 nm thermally grown silicon oxide (SiO_2_); the main process steps are shown in Figure 2. Prior to processing, the wafer was cut into (2 × 2) cm² squares, which were treated with 100% isopropyl alcohol in a sonicator for 2 min. A 5–10 nm thick nickel (Ni) layer was deposited via sputtering (PlasmaLab System 400, Oxford Instruments, Abingdon upon Thames, UK) to facilitate the adhesion of silver (Ag) to the substrate. Subsequently, a silver layer (Ag, 99.99% pure, Kurt J. Lesker Company Ltd., East Sussex, UK) with thickness h = 25–125 nm was deposited using thermal evaporation at a rate of 0.15 nm/s in a vacuum of 1 × 10^−6^ Torr. A 250 nm thick polymethyl-meth acrylate (PMMA; 4% 950 K, 679.04, Allresist GmbH, Strausberg, Germany) layer was deposited as the e-beam resist, which is structured using an electron beam lithography system (Pioneer, Raith GmbH, Dortmund, Germany) within a total area of 1.2 mm² to write grating structures with periodicities ranging from Λ = 700–900 nm. After developing the e-beam resist, a second silver deposition step using the same parameters was performed, such that the Ag base layer thickness (b) and the grating structure height (h) could be adjusted. In this study, only symmetric gratings with w = g were fabricated. The final lift-off process used pure acetone in a sonicator for 15 min, followed by a cleaning step in pure isopropanol (IPA) for 2 min. In total, six different periodicities Λ, each with four different heights (h) and varying base thicknesses (b), were produced in order to study the respective effects on the performance and improve the simulation model. The resulting structures were characterized with the additional help of optical and scanning electron microscopy (SEM) images.

**Figure 3:**
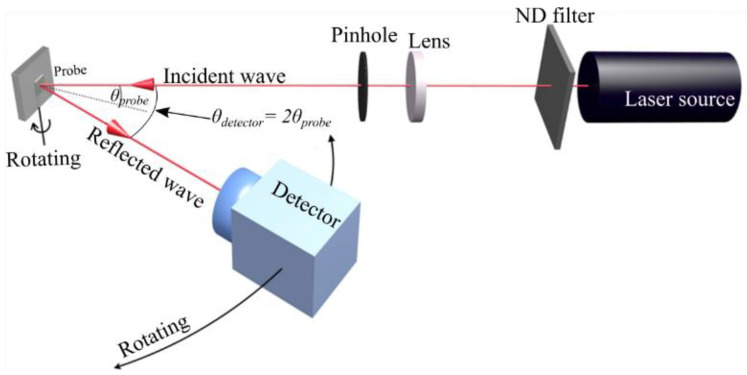
Diagram of the measuring setup shows the position of the sample and detector in the same plane. The setup automatically rotates the detector section such that the reflection is recorded. The adjustment of the beam’s polarization and the grating structure were set such that the azimuthal angle is 90°.

To characterize the grating structures’ performance, surface plasmons using the grating samples were excited using a dedicated home-built measurement setup, which is depicted in Figure 3. The polarization of the light output of a 632.8 nm helium–neon gas laser (05-LHP-151, Melles Griot, Singapore) was adjusted using an integrated polarizer to provide a linearly polarized beam.

The beam’s intensity was set to 1 mW using a neutral density filter (ND filter). The near-ideal Gaussian beam output was collimated with a diameter of 3 mm. In order to further improve the beam profile, the light was focused onto a pinhole using a plano-convex lens with a focal length of f = 300 mm to block any higher order Gaussian mode contributions. It was subsequently collimated again to obtain a beam diameter of 1 mm in order to ideally illuminate the grating structure during the experiments. The corresponding sample holder was designed for the automatic reflection characteristic recording from 10 to 40 degrees, with a 0.2-degree angular resolution. To gauge the coupling efficiency, the reflected beam intensity for varying excitation angles was recorded. To this end, a light detector (ANDO, Ando Electric Co., Ltd., Tokyo, Japan) simultaneously rotated at twice the sample angle.

To conduct the biological tests, a solution of PBS (phosphate-buffered saline) was prepared, which included antibody human immunoglobulin G; IgG (150 kD, I4506, Sigma-Aldrich, Saint Louis, MO, USA) and protein A (42 kD, P6031, Sigma-Aldrich). The IgG solution with a concentration of 5 µM (0.75 mg/mL) was carefully applied to the grating structure using a micropipette. A total volume of 5 µL was deposited using a damp ring with a diameter of 2.5 mm placed around the grating area, which allowed for a reproducible production of the analyte layers. Subsequently, the solution was left to dry. This ensured that the biological samples came into contact with the grating surface. Once the solution dried on the grating, the plasmon excitation angle was measured using the same experimental setup described earlier. This initial measurement provided an IgG signal for comparison. Next, a second solution containing protein A at a concentration of 10 µM (0.42 mg/mL) was dropped with the same method described above onto the previously applied IgG solution. This step facilitated the physical binding of protein A to IgG on the grating surface. After the second solution dried, the plasmon excitation angle was scanned again using the experimental setup. This additional scanning allowed for the characterization of the interactions between the IgG and protein A on the grating structure. By comparing the plasmon excitation angles obtained before and after the protein A binding step, the influence of protein A on the optical properties of the grating structure could be assessed. These measurements provide insight into the physical interactions and effects induced by the binding of protein A to IgG on the grating’s surface. This experiment focused on demonstrating the feasibility of using a grating as a biosensor for real samples. The goal is to show that the grating could interact with the biological sample and generate a detectable response, highlighting the potential application of the grating as a biosensor. In order to evaluate the long-term stability, the grating structures were repeatedly characterized after fabrication and after 1 week, 5 weeks, and 8 weeks, respectively. Different protective coatings were applied in order to enable a study regarding their effect on aging and the long-term stability of the grating coupler structures. In particular, 20 nm of SiO_2_ and silicon nitride (Si_3_N_4_) were deposited using a plasma enhanced chemical vapor deposition (PECVD) technique, respectively.

In parallel, the grating model was further developed by comparing the simulation results with the experimental results and analytically calculated values. The simulation model was built in Matlab based on a previous work [17]. The conventional finite-difference time-domain (FDTD) method [18] was applied for the investigation of reflectivity onto the grating structure. Although silver is the predominant metal of the grating structure, the nickel layer was considered in the simulation, assuming a thickness of 10 nm. The parameters for the silver base are given as a thickness (b) of 100 nm and a grating height (h) of 50 nm; the silver width (w) and the gap width (g) were adjusted in the simulations according to the respective achieved experimental values. For the purpose of establishing a relationship between the plasmon excitation angle and the grating period, they were set w = g within the range of 350–450 nm, which is a grating period of 700–900 nm. Additionally, for simulating the sensor with various refractive indices, the grating size was specifically set to w = g = 400 nm, which is a grating period of 800 nm. The permittivity values of nickel and silver were modeled at a wavelength of 632.8 nm with −11.4 and −19, respectively.

Overall, the focus of this work was placed on the optimization of the coupler structure via varying layout parameters, including the size and periodicity of the coupling structure, and characterizing the effect these parameters have on the angle at which the maximum coupling occurs. The main effort was investigating the properties of the metals, as well as the propagation and excitation of the wave.

## 3. Results

### 3.1. Experimental Characterization of Grating Performance

**Figure 4:**
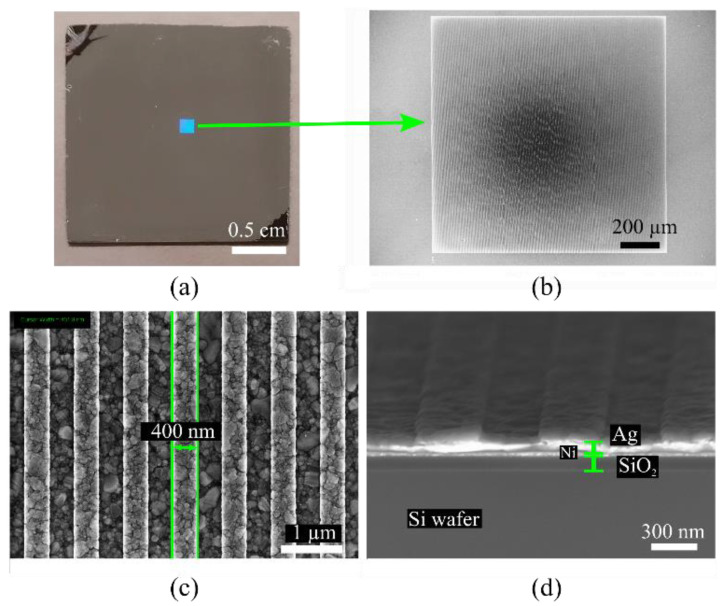
(**a**) The grating structure on the wafer size of (2 × 2) cm^2^. (**b**) The complete grating area size of (1.2 × 1.2) mm^2^ was captured by scanning electron microscope. (**c**) Silver grating size of 400 nm, i.e., a grating period of approximately 800 nm. (**d**) Cross-section of grating structure.

A visual evaluation of the grating structures was performed to verify the resulting morphology and grating parameters. Figure 4 depicts the size of the actual grating area on the silicon substrate (2 × 2) cm^2^ and the silver surface using electron microscopy.

**Figure 5:**
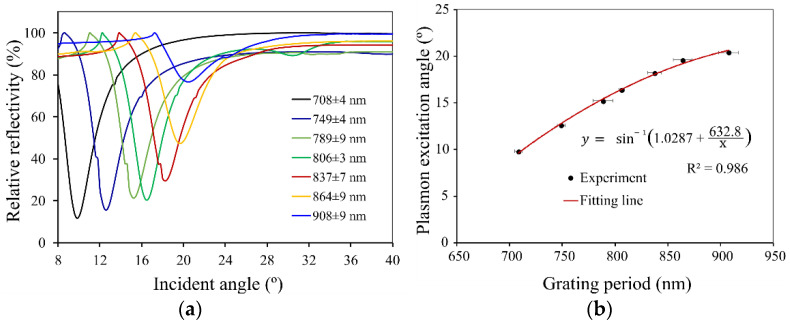
(**a**) Relative reflectivity of the silver grating with a base thickness of 100 nm and a grating height of 50 nm in the variation of the grating period from 700–900 nm. (**b**) Relation of plasmon excitation angle and grating period.

Subsequently, the plasmon wave excitation was investigated for the different grating parameters. The influence of the periodicity Λ on the angle at which the maximum absorption of the light takes place was determined, as well as the full width half-maximum (FWHM) peak width and the peak amplitude. The characterization results for a silver base thickness of b = 100 nm and grating height h = 50 nm are summarized in Table 1 and shown in Figure 5. The error in determining the periodicity was calculated via SEM picture analysis.

Accordingly, the results were used to evaluate the grating’s ideal coupling angles as a function of the spatial period, as shown in Figure 5b. A curve fit using Equation (1) was used to determine the fitting parameter $\alpha =\frac{1}{n_{a}}\sqrt{\frac{\epsilon_{m\;}\epsilon_{a}}{\epsilon_{m}+\epsilon_{a}}}$ and yielded a coefficient of determination 𝑅^2^ = 0.986. The resulting value of α=1.0287 is aligned closely with the theoretical expectations when using the experiment’s theoretical values for $n_{a\;}=1,\;\epsilon_{m\;}=-15.243,\;\epsilon_{a}=1$ to estimate $\alpha_{theory}=1.0345$. The experimental data obtained thus demonstrate a strong agreement with the underlying theory.

The influence of the grating height (h) was first investigated at a constant base thickness b = 100 nm. For this purpose, couplers were produced with heights (h) between 25–100 nm, which were compared to each other as a function of the reflection and the intensity of the reflected light. The range of the fabrication variation was considered depending on the preliminary results of previous work. The range of the angle that the optical measurement setup can perform is 10°–40°, which covers the anticipated coupling angles of all the structures produced.

The behavior of a relative reflectivity curve varies. Some of the curves showed a shallow minimum at the maximum absorption angle; therefore, several dip angles were not plotted with a full profile. The data details below 10° could not be determined with high precision due to the measuring setup design limitations. The range of angles between 15°and 20° is the most relevant for the structures produced in this study. However, the design of the maximum absorption angle should start with a small angle because when the sensing layer changes, the maximum absorption will increase. For this reason, the small angle of the baseline angle will have a more ample detection range.

**Figure 6:**
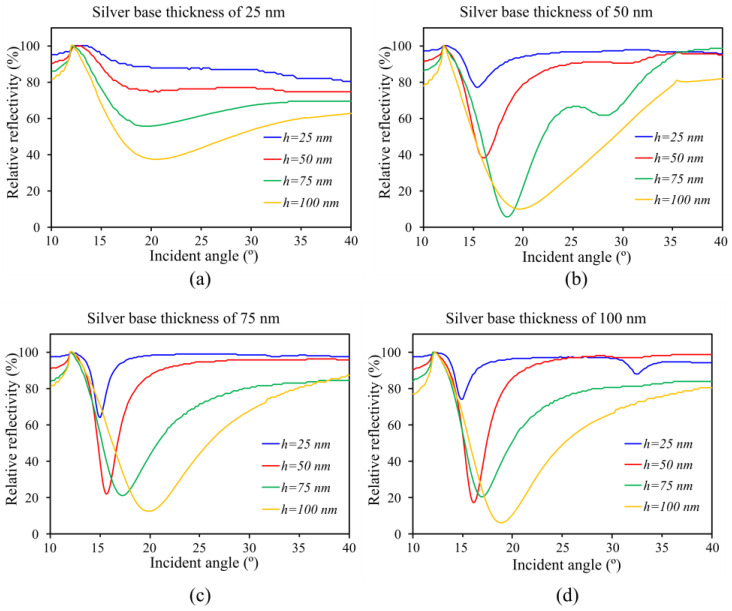
Relative reflectivity (%) of the grating for different silver base thicknesses and grating heights; the incident angle was scanned from 10°–40°. The grating structures on the silver base were examined with thicknesses of 25 nm (**a**), 50 nm (**b**), 75 nm (**c**), and 100 nm (**d**), along with grating heights (h) of 25 nm, 50 nm, 75 nm, and 100 nm, respectively. Among these, the grating structures with a silver base thickness of 75 nm and 100 nm, combined with a grating height of 50 nm, exhibited the highest performance, characterized by lower relative reflection and a smaller FWHM value.

A total of 16 samples were compared with the same grating size of 400 nm and the period of 800 nm with the variation of silver-based thickness b = 25–100 nm and grating height h = 25–100 nm. For this fabrication series, the samples’ analysis is provided with a fixed thickness of a base layer or a grating height. The performance of the samples can be characterized by the absorbance strength and the full width at half-maximum (FWHM) of the absorption curve signal [19] at the excitation angle. Moreover, the power at the maximum absorption angle was compared with 100% power of the reflected wave, which represents the efficiency of the grating coupling. Figure 6a–d shows that the thickness of the silver base and grating height had an important impact on the plasmon behaviors at different levels.

The coupling efficiency (CE) was evaluated through calculations based on the characteristics of the signal, namely, the relation between the full width at half-maximum (FWHM) of the relative reflectivity δR and relative reflectivity $R_{rel}$, according to Equation (2):

(2) $$\mathrm{CE}=\frac{100-R_{rel}}{\delta R}$$

The reduced spectral broadening and lower reflectivity contributed to a higher proportion of incident light being coupled into the grating, resulting in higher efficiency, which can be appreciated in Figure 6. The base layer’s thicknesses exceeding 75 nm did not affect the excitation angle.

On the other hand, the grating height shows a sharp signal as small FWHM at 50 nm of the grating height, but it is less sharp when the grating is higher than 75 nm.

**Figure 7:**
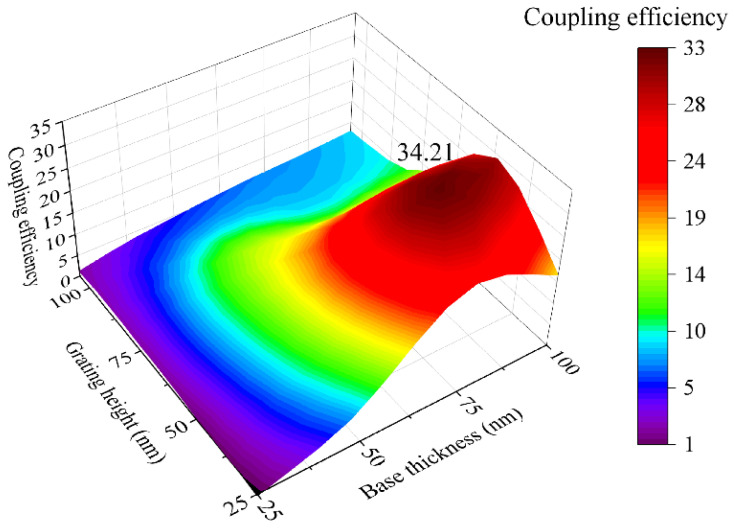
Coupling efficiency in grating structures with varying base thicknesses and grating heights, from 25 to 100 nm. The calculations were conducted considering a signal characterized by the full width at half-maximum (FWHM) and relative reflectivity. The highest coupling efficiency by the contour graph is 34.21, achieved from the base thickness of 84 nm and grating height of 44 nm.

It is shown by the results that a grating base layer with a thickness b = 75–100 nm and grating height of h = 50 nm yield the best performance for silver-based gratings. The grating’s structural parameters are highly significant for sensing purposes, and a graphic representation of the coupling quality versus the structural parameters of the grating is provided in Figure 7 using interpolation of all the experimentally obtained values.

While the experiment results achieved the highest coupling efficiency of 32.50 with structures with a base thickness b = 75 nm and height h = 50 nm, the data analysis points towards an ideal coupling efficiency of 34.21 when producing structures with a base thickness b = 84 nm and grating height h = 44 nm. Consequently, the experimentally obtained coupling efficiency is within 5% of the ideal value.

### 3.2. Iterative Simulation Optimization

**Figure 8:**
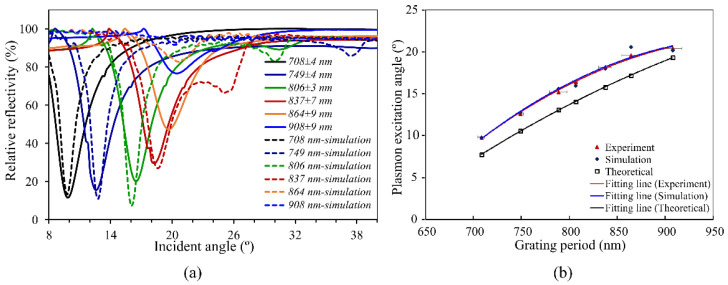
Comparison of experimental results and simulation results. (**a**) The dashed lines represent simulation results, and solid lines are the experiment results. (**b**) Comparing the three results, the experiment and simulation showed remarkably similar outcomes; however, the theoretical calculation yielded a lower number.

The ratio of the respective penetration depths in the simulation corresponds to the experimental results. It is important to note that in order to determine the absolute penetration depth, the reflectivity was assessed somewhat differently in the simulation than in the experimental case. Only the power reflected at the angle of incidence was determined in the latter case. In contrast to this, the power reflected over the entire angular range was used in the simulation to determine the reflectivity. The curve in Figure 8b shows the line of the results of the experiment, simulation, and ab initio calculation based on Equation (1).

Overall, the values determined by the simulation agree with both the theoretical and experimental values. In particular, the sensitivity parameter, which is important for evaluating sensors, was reproduced by the simulation. The experiment and simulation show good agreement, indicating that the simulation accurately represents the real-world behavior of the phenomenon. The close correspondence between these results indicates the reliability of the simulation model. However, the theoretical calculation resulted in slightly lower values, which is likely due to simplifying assumptions or limitations in the theoretical model. It is important to note that the theoretical calculations are based on idealized conditions and may not perfectly align with real-world observations.

In order to obtain a simulation model resembling real-world behavior to a high degree, the model was adjusted to fit to the experimental works based on the coefficient and refractive index of the materials. After modification, the model was used for the sensor propose to analyze the sample over the grating structure in various refractive indexes.

In the literature, the refractive indexes (RIs) of biological samples have been reported in theoretical and experimental studies to range from 1.33 to 1.46. The approximate values of the biological samples from previous reports are summarized in Table 2.

**Figure 9:**
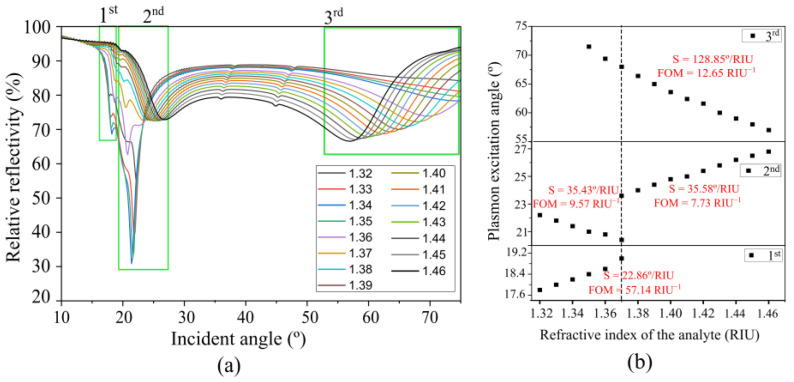
(**a**) Simulation of the reflectivity of the sensor with various refractive indices of the analytes and the various diffraction orders. (**b**) Resonant angle as a function of refractive index. The behavior of the plasmon excitation angle for varying refractive indices is dependent on the order of the diffraction employed for sensing.

They have been tested in various models, such as protein, DNA, intra- and extra-cellular fluid, etc. Therefore, this study has simulated and tested situations covering the range of RIs of the most recent biological sensors studies. Figure 9 shows that the proposed sensor could be a potential candidate for sensing the analyte of two refractive indexes ranging from 1.32 to 1.37 and 1.37 to 1.46.

According to Equation (1), *θ* varies with refractive index *n_a_*, thus the grating configuration can be used as a refractive index sensor. The sensor’s sensitivity *S* is defined as follows:

(3) $$S=\frac{\Delta \theta }{\Delta n_{a}}$$

where Δ*θ* is the change in the plasmon excitation angle corresponding to Δ*n_a_* change in the analyte refractive index. The figure of merit (FOM) is another measure used to quantify sensor performance [26]. It is defined as FOM = S/FWHM, where FWHM is the full width at half-maximum of the absorption curve signal at the excitation angle. A higher *S* and a larger FOM can be used as sensor performance parameters.

The SPR response of the sensor with various refractive indexes of the analytes are shown in Figure 9a. The first range of the refractive index varied from 1.32 to 1.37. It was found that the dip strength was about 30–70%. The plasmon excitation angle as a function of the refractive index demonstrated a positive trend for the first diffraction order, but showed a negative trend for the second diffraction order, as shown in Figure 9b. The sensitivity of the proposed sensor is 22.86°/RIU (FOM = 57.14 RIU^−1^) and 35.43°/RIU (FOM = 9.57 RIU^−1^) for the first and second diffraction orders, respectively. The second range of the refractive index varied from 1.37 to 1.46. Unlike the study conducted on the refractive index range of 1.32 to 1.37, the result only shows the presence of the second diffraction order and the third diffraction order. The results show the sensitivity of sensor 35.58°/RIU (FOM = 7.73 RIU^−1^) and 128.85°/RIU (FOM = 12.65 RIU^−1^) for the second and third diffraction orders, respectively. A comparison between this study and previously reported works is also given in Table 3.

### 3.3. Sensing Performance and Long Term Stability

**Figure 10:**
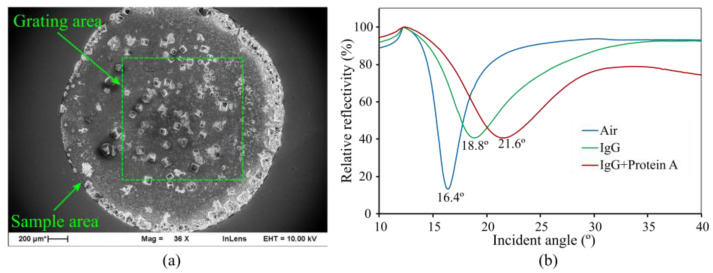
(**a**) SEM detail of the microscope morphology of antibody (IgG) and protein A on the grating structure. (**b**) Relative reflectivity (%) of the grating for air (16.4°), antibody (IgG) (18.8°), and protein A bound to immobilized IgG antibody (21.6°); the incident angle was scanned from 10° to 40°.

The results that were obtained for the examination of biomolecules using antibody-based techniques are presented in Figure 10.

The experiment tracked the changes that occurred during each step of the process. Figure 10a shows an electron microscope image of a sample on the grating structure; it showcases the success of the drop solution on the grating area. The sample area effectively covered the entire grating structure, with the observed morphology indicating the presence of dry biosamples. Notably, the image reveals the occurrence of the coffee-ring effect [27], where particles tend to concentrate along the original drop edge. However, due to the drop area being larger than the grating area, this effect was mitigated, reducing its impact on the experiment, while Figure 10b illustrates the relative reflectivity chart with respect to the plasmon excitation angle. The initial measurement involved analyzing an air sample to ensure that the grating structure was properly constructed and functioning. The result serves as the baseline, indicating a plasmon excitation angle of 16.4° for air as the analyte. Subsequently, the maximum plasmon excitation angle increased to 18.8°, reflecting the presence of IgG molecules. In the next step, a protein A solution was applied as a second layer onto the grating structure, allowed to dry, and then measured again. The results revealed a further shift in the plasmon excitation angle to 21.6°, indicating the influence of protein A on the grating structure. The reflectivity curve for IgG + protein A demonstrates a pronounced impact of protein A association on the curve, particularly noticeable by the considerable decrease in the baseline reflectance at high incident angles. This effect can be attributed to two potential explanations. Firstly, the thickness of the analyzed layer, which encompassed the immobilized IgG and associated protein A, is likely greater than that of the IgG-only layer, thereby influencing the signal profile (refer to Figure 6, specifically when h > 75 nm). Secondly, the combination of IgG and protein A might exhibit a secondary deep angle near 40°, potentially leading to interference with the baseline reflectance (as depicted in Figure 10b). These factors collectively contribute to the notable decline in the baseline reflectance observed at high incident angles.

Furthermore, referring to the simulation results in Figure 9, it was calculated that the refractive index of the IgG layer was 1.367, while that of the IgG + protein A layer was 1.337. These values provide additional insight into the optical properties of the respective layers in the grating structure. In this specific experiment, the interaction between protein A and IgG did not occur with high affinity or in a strictly controlled manner; however, it is important to clarify that the drop-on surface method primarily focuses on demonstrating the concept of refractive index changes rather than conducting a kinetic study of physical binding.

**Table 3:** Comparison of sensor preferment with other previous works.

Structure Configuration	Method	Coating	Wavelength (nm)	Sensitivity (°/RIU)	References
Prism	Sim.	Ag-Au	632.8	54.84	[28]
Prism	Sim.	Graphene-hybrid	633	56.34–60.62	[29]
Prism with air gap	Sim.	Ag-graphene	633	61.54–68.03	[30]
Grating integrated prism	Sim.	Ag-graphene	633	220.67	[31]
Bimetallic grating	Sim.	Al-Au	900	187.2	[32]
Bimetallic grating	Sim.	Ag-Au	920	346	[33]
Two-dimensional bimetallic alloy grating	Sim.	Ag, Au, Cu, Pd, Pt	700	152–161	[34]
Grating with phase-interrogation	Exp.	Au	633	300	[35]
Multilayer grating	Sim.	Al-Au	900	279.6	[36]
Grating	Exp./Sim.	Ag	632.8	22.86–128.85	This work

Simulation (Sim.), experiment (Exp.).

**Figure 11:**
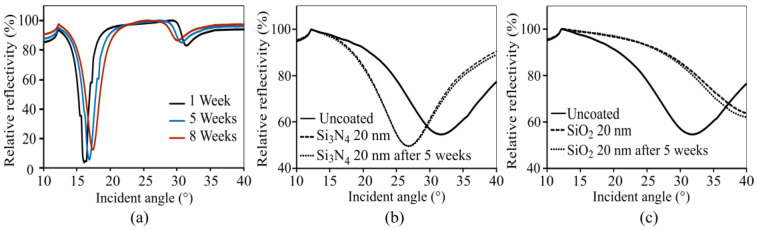
(**a**) Without protective coating, the excitation angles drifts considerably over time due to changes to the silver coating material. (**b**) Using Si_3_N_4_, the excitation angle shifts to smaller angles, but the performance of the grating coupler remains constant over time. (**c**) The use of SiO_2_ shifts the excitation angle to higher values and the long-term stability is inferior when compared to using Si_3_N_4_.

The results of the long-term stability test with and without protective coatings is depicted in Figure 11.

The unprotected silver grating structure quickly deteriorated within a few weeks, leading to a shift in the resonance angle and coupling efficiency. The use of protective layers shifts the respective excitation angles according to changes in the effective grating periodicity due to altered refractive indices. The Si_3_N_4_ layer did not show any drifts, while the SiO_2_ layer led to a slower but considerable drift. Even though SiO_2_ and Si_3_N_4_ have emerged as suitable platforms for a protective layer, silicon functionalization must be carefully considered. Physical absorption was used in this study as a simple approach to immobilizing biomolecules on silicon oxide. However, other approaches have been refined to achieve maximal surface coverage while minimizing biomolecule activity loss [37]. Covalent bonding [38,39] has demonstrated excellent stability and long-term immobilization capabilities. Self-assembled monolayers (SAMs) [40,41] provide controlled surface modification and enhanced specificity. The sol–gel method [42] offers versatile coating possibilities and excellent biocompatibility. Considering these techniques is crucial for successfully translating silicon-based immobilization strategies into real-world applications.

## 4. Discussion

The interpretation of the experimental results of the grating parameters is twofold: Firstly, a precise control of the structure parameters is mandatory in order to produce reproducibly performing grating structures. Secondly, the underlying theory describing the coupling behavior is incomplete since it is not able to take into account the effect of the base layer thickness and composition. The experimental results strongly indicate that the thickness of the metal layer is a main factor influencing the coupling efficiency. Previous works by other groups have illustrated similar behavior [31,43] and have also reported on the effects that the base thickness and the grating’s spatial period have on the excitation angle of the surface plasmons and the full width at half-maximum (FWHM) of the signal. Herein, the ideal parameters have been determined from the experimental results.

Likewise, the experimental results have been used to fine-tune a simulation model to provide a precise computer-based tool to evaluate the design of tailor-made structures. The deviation of the ab initio calculation from the simulation results indicates that the theoretical description of plasmon excitation via grating structures is incomplete and a more comprehensive description that considers the base layer thickness is required. Overall, the data obtained from these experiments and simulations highlight the changes in the plasmon excitation angles and reflectivity, demonstrating the impact of the biomolecules on the grating structure and enabling the characterization of their performance.

Lastly, the experiments using real-world samples demonstrate the potential of using silver-based grating structures for biosample analysis, as well as the broad range of refractive index changes that may be monitored. The results on the long-term stability highlight the necessity to investigate methods to extend the lifetime of silver-based structures. While recovering the performance via chemical or thermal treatments are possible routes to enhance the lifetime of the grating coupler, the use of a protective layer is a viable alternative. In addition, the changes in behavior of both the plasmon excitation angle and the coupling efficiency show the need to further expand the theoretical description and simulation model in order to provide comprehensive means for designing SPR-based biosensors.

## 5. Conclusions

The grating coupler was fabricated on a silicon wafer using silver as highly sensitive plasmonic material. The parameters of the grating structure influenced the plasmon excitation angle. The experimental results have shown that the silver-based grating structure configuration with a grating period Λ = 800 nm, a silver-based thickness of b = 75–100 nm, and a grating height of h = 50 nm yielded the best sensitivity. Moreover, the simulation model was built to predict the characteristics of the grating structure for all the relevant experimental parameters. The computer model performed the related results with the experimental results in the range of Λ = 700–900 nm for the grating period. Therefore, the model should improve the parameters to be viable in future work. For the purpose of sensing, the grating SPR performance within the RIs range of 1.32 to 1.46 showed two different trends. The first range of 1.32 to 1.37 showed sensitivity of about 22.85, 35.43°/RIU and the second range of 1.37 to 1.46 was 35.58, 128.85°/RIU.

In the biological sample, the simulation results and experimental results reveal the refractive indices of the IgG and IgG + protein A layers within the grating structure. The IgG layer had a refractive index of 1.367, while the IgG + protein A layer had a refractive index of 1.337. These values provide insight into the optical properties of the respective layers and their interaction with light within the grating structure. The use of a protective 20 nm thick Si_3_N_4_ layer proved to be an effective means to introduce long-term stability into silver-based grating structures.

In addition, the results show that the grating period, the thickness of the grating, and the grating base are key parameters in improving the resolution and sensitivity of the grating sensor. The grating period can be an indicator for selecting the angle of the incident to excite plasmons. Additionally, the thickness of the base and grating height can improve the resolution and sensitivity by showing the FWHM. Therefore, to achieve the improvement of plasmonic grating sensing, three parameters must be considered before using it.

## Figures and Tables

**Table 1:** Variation of grating period size.

Grating Period (nm)	SD (nm)	Excitation Angle (°)	Relative Reflectivity (%)	FWHM (°)
708	4	9.8	11.58	5.2
749	4	12.6	15.32	3.8
789	9	15.2	21.31	3.4
806	3	16.4	20.16	3.6
837	7	18.2	29.44	3.2
864	9	19.6	47.40	3.8
908	9	20.4	76.74	3.2

**Table 2:** List of approximate values for refractive index of biological samples.

Medium	Refractive Index	References
Water, PBS	1.33, 1.332	[20]
Extra- and intra-cellular fluid	1.34–1.35, 1.35–1.36	[21]
Proteins, lipids, DNA	1.40, 1.42, 1.44	[22]
Skin, muscle, adipose	1.36, 1.39, 1.46	[23]
Blood plasma	1.335	[24]
Hemoglobin	1.354	[25]

## Data Availability

The data underlying the results presented in this paper are not publicly available at this time but may be obtained from the authors upon reasonable request.
